# Effect of changes in skin properties due to diabetes mellitus on the titration period of transdermal fentanyl: single-center retrospective study and diabetic animal model study

**DOI:** 10.1186/s40780-024-00402-5

**Published:** 2024-12-18

**Authors:** Satoshi Mizuno, Makiko Takabayashi, Hiroko Makihara, Kazuhiro Ogai, Kei Tsukui, Yuriko Ito, Takahiro Kawakami, Yusuke Hara, Arimi Fujita, Yoshihiro Tokudome, Tomoko Akase, Yukio Kato, Tsutomu Shimada, Yoshimichi Sai

**Affiliations:** 1 Department of Clinical Pharmacokinetics, Graduate School of Medical Sciences, Kanazawa University, 13-1 Takara-Machi, Kanazawa, 920-8641 Japan; 2 Department of Hospital Pharmacy, University Hospital, Kanazawa University, 13-1 Takara-Machi, Kanazawa, 920-8641 Japan; 3 Department of Biochemistry, School of Medicine, Tokyo Women’s Medical University, Tokyo, Japan; 4 Department of Biological Science and Nursing, Graduate School of Medicine, Yokohama City University, Yokohama, Japan; 5 Department of Bio-Engineering Nursing, Graduate School of Nursing, Ishikawa Prefectural Nursing University, Kahoku, Japan; 6 The United Graduate School of Agricultural Sciences, Kagoshima University, Kagoshima, Japan; 7 Laboratory of Cosmetic Sciences, Institute of Ocean Energy, Saga University, Saga, Japan; 8 Department of Molecular Pharmacotherapeutics, Faculty of Pharmacy, Kanazawa University, Kanazawa, Japan; 9 Department of Clinical Pharmacy and Healthcare Science, Faculty of Pharmacy, Institute of Medical, Pharmaceutical and Health Science, Kanazawa University, Kanazawa, Japan; 10 AI Hospital/Macro Signal Dynamics Research and Development Center, Institute of Medical, Pharmaceutical and Health Sciences, Kanazawa University, Kanazawa, Japan

**Keywords:** Transdermal fentanyl, Opioid titration, GK rats, Diabetes mellitus, Stratum corneum, Intercellular lipids, Ceramides

## Abstract

**Background:**

In the dose titration of transdermal fentanyl to prevent unrelieved pain, it is important to consider not only dose adjustment, but also the titration period, which is influenced by the time required to reach the steady state. Many patients with cancer pain experience comorbidities that might affect the skin properties and influence transdermal absorption. We hypothesized that skin changes due to diabetes mellitus (DM) would affect the titration period of transdermal fentanyl. We conducted a retrospective study and diabetic animal model study to test this hypothesis.

**Methods:**

In the retrospective study, the titration period was defined in terms of “dose change” and “number of rescue opioids” in patients initiated on transdermal fentanyl. Multiple logistic regression analysis was performed to analyze the relation between the titration period and comorbidities, including DM. In the diabetic animal model study, intercellular lipids of stratum corneum (SC) were analyzed in Goto-Kakizaki (GK) rats, a model of DM, and the pharmacokinetics of intravenously or transdermally administered fentanyl was examined.

**Results:**

In the retrospective study, the titration period ranged from 5 to 39 days (n = 387), and the patients taking a longer period (6 days or more) was significantly related to in patients with unspecified DM: AOR (95% confidence interval), 0.438 (0.217–0.884). In the diabetic animal model study, the ceramides (CERs) content in the SC was decreased by approximately 30% in GK rats compared to Wistar rats. The absorption rate constant (*k*_a_) of fentanyl administered transdermally was increased approximately 1.4-fold in GK rats, though there was no difference in transdermal bioavailability (*F*) or systemic clearance (*CL*_tot_).

**Conclusion:**

Our results suggest that the steady state of transdermally administered fentanyl is reached sooner in cancer patients with DM as a comorbidity. Earlier pain assessment and dose adjustment may be possible in these patients.

**Supplementary Information:**

The online version contains supplementary material available at 10.1186/s40780-024-00402-5.

## Background

Transdermal fentanyl relieves moderate to severe cancer pain and chronic pain [1, 2]. Unrelieved pain impacts all dimensions of quality of life [3], so needs to be controlled rapidly after switching from other opioids to transdermal fentanyl. However, due to the opioid-specific adverse effects [1, 2] and a large inter-individual variability in drug release from the transdermal formulation [4, 5], the dose must be carefully titrated. Therefore, factors related to transdermal fentanyl dose at the steady state, including gender, age, body mass index (BMI), and serum albumin, have been investigated [6–10], but so far there is little information on factors affecting the titration period.

There is a relationship between blood fentanyl concentration and the extent of pain relief [11, 12], and the dose titration period of transdermal fentanyl is influenced by the time required for the blood concentration to reach a steady state. The disposition of transdermal fentanyl is described by a one-compartment distribution model with first-order absorption and elimination, and follows flip-flop kinetics [13–16], where the half-life depends on the absorption rate constant (*k*_a_). The rate-limiting step in transdermal absorption is diffusion through the stratum corneum (SC), the outermost layer of the skin [17, 18]. The SC consists of corneocytes embedded in a lipid matrix, which acts as the main barrier to diffusion of substances through the skin [19].

More than half of cancer patients have at least one comorbidity [20, 21]. Cancer and diabetes mellitus (DM) are prevalent globally and are frequently diagnosed in the same individual [22, 23]. In addition, many diabetic patients experience cutaneous complications [24], such as diabetic ulcers due to impaired healing [25]. Furthermore, diabetic animal models, such as Otsuka Long-Evans Tokushima Fatty (OLETF) rats and db/db mice, show decreased levels of intercellular lipids in the SC even in non-injured skin [26, 27]. Such lipid changes may affect the permeation of drugs.

We hypothesized that skin changes due to comorbidities, including DM, would affect the dose titration period of transdermal fentanyl. To test this hypothesis, we carried out a retrospective study in cancer patients and a diabetic animal model study to investigate the titration period and the pharmacokinetics of transdermal fentanyl.

## Methods

### Retrospective study

**Fig. 1:**
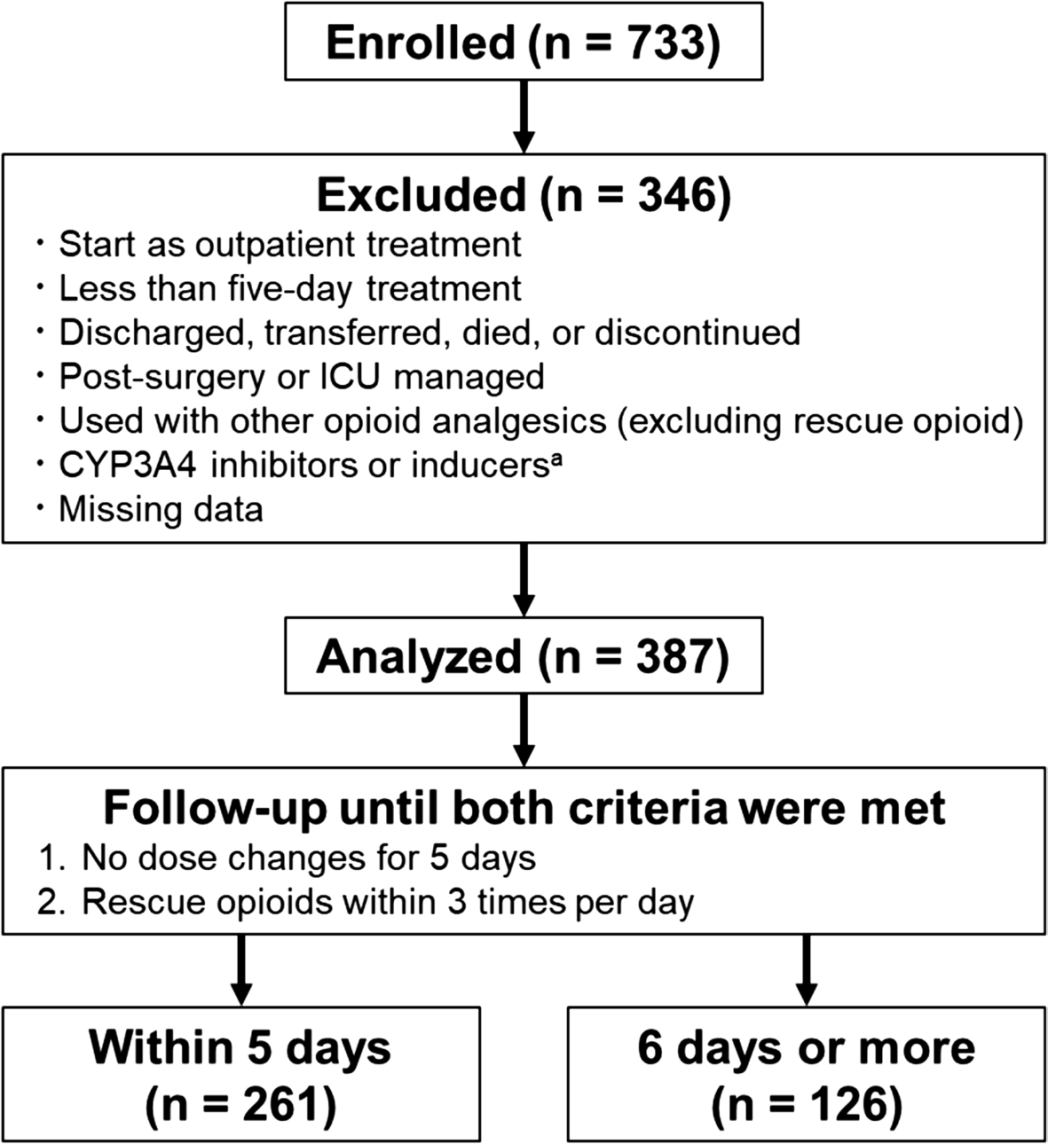
Flowchart of analysis in the retrospective study. In total, 733 patients were enrolled, and 387 patients were analyzed. Among them 261 patients showed a titration period of within 5 days, and 261 showed a titration period of 6 days or more. ^a^ Inducers or inhibitors of CYP3A4 used in excluded patients were as follows: carbamazepine, enzalutamide, mitotane, phenobarbital, phenytoin, rifampicin, amiodarone, cobicistat, clarithromycin, diltiazem, fluvoxamine, indinavir, itraconazole, nelfinavir, saquinavir, ritonavir, and voriconazole

Patients who initiated treatment with 24 h-fentanyl citrate formulation (Fentos tape) between Apr 1, 2015, and Mar 31, 2019, at Kanazawa University Hospital were enrolled. Exclusion criteria are shown in Fig. 1. The survey items were gender, age, height, weight, BMI, serum albumin (before treatment), comorbidities listed on the physician's problem list (including history), initial dose of transdermal fentanyl, and the titration period. Comorbidities were coded using the International Classification of Diseases 10th Revision (ICD-10) ver. 2014 three-character categories (A00-Q99). For cancer metastases, four-character subcategories were used (C78.0-C79.9). The titration period was defined as the time until two criteria, “no dose change for 5 days” and “rescue opioids within 3 times per day” were both met. The half-life of fentanyl after removal of the transdermal formulation is 21.9 ± 8.9 h (mean ± S.D.) [28], so that the steady state should be reached in about 5 days. Furthermore, the European Society for Medical Oncology (ESMO) guidelines recommends baseline opioid adaptations if rescue opioids are needed more than 4 times per day [2].

### Chemicals

Fentanyl citrate injection and fentanyl citrate formulation (Fentos tape) were purchased from Terumo Corporation (Tokyo, Japan), and Kyowa Kirin Co., Ltd. (Tokyo, Japan), respectively. Isoflurane was purchased from Viatris Inc. (Canonsburg, PA, USA). Epilat hair removing cream was purchased from Kracie Ltd. (Tokyo, Japan). Trypsin from bovine pancreas was purchased from Sigma-Aldrich Co. LLC (St. Louis, MO, USA). Norfentanyl-D5 oxalate and papaverine hydrochloride were purchased from Sigma-Aldrich Co. LLC (St. Louis, MO, USA) and Nacalai tesque (Kyoto, Japan), respectively. All other chemicals were of analytical or high-performance liquid chromatography (HPLC) grade.

### Animals

Goto-Kakizaki (GK) rats as a diabetic animal model [29] and Wistar rats as controls were purchased at 10 weeks old from Japan SLC (Hamamatsu, Japan). GK rats were fed a low protein/high carbohydrate diet (LABO MR-DBT, Nosan Corporation, Yokohama, Japan) and Wistar rats were fed a standard diet until experiments at 12–14 weeks old. Diets were provided ad libitum. Non-fasting blood glucose levels were measured using Glutest Neo Alpha (Sanwa Kagaku Kenkyusho, Nagoya, Japan). The hairs on the lateral abdominal skin were removed by treatment with hair removal cream at least 1 h before the experiments.

### Analysis of skin parameters and intercellular lipids of SC in rats

Transepidermal water loss (TEWL), SC hydration, and surface pH in the lateral abdominal skin were measured under isoflurane anesthesia using a Tewameter TM300MP, Corneometer CM825, and Skin-pH-Meter PH905, respectively (Courage + Khazaka electronic GmbH, Köln, Germany). Each value was calculated as the mean of three individual measurements. The SC sheet was separated from the lateral abdominal skin by treatment with 0.1% (w/v) trypsin in water for 21 h at 4ºC and 5 h at 37ºC. Free fatty acids (FFAs), ceramides (CERs), and cholesterol (CHOL) in the SC were measured as described previously [30]. CERs was calculated for a total of 12 CER subclasses [19].

### Intravenous and transdermal administration of fentanyl in rats

For intravenous administration, fentanyl citrate injection diluted in saline (10 µg fentanyl/2 mL/kg body weight) was injected via the jugular vein over a period of 10 s under isoflurane anesthesia. Blood samples (100 µL) were collected from the jugular vein using a heparin-coated syringe at 1, 5, 10, 20, 30, 60, 120, 180, and 300 min. For transdermal administration, one-fourth of 1 mg fentanyl citrate formulation (160 µg fentanyl) was applied to the lateral abdominal skin and blood samples were collected at 1, 2, 4, 8, 12, 24, 30, 48 h. The formulation was removed at 24 h and stored at 4ºC until analysis. The blood samples were centrifuged, and plasma samples were stored at -80ºC until analysis.

### Analysis of fentanyl in the blood samples and the formulation

Plasma fentanyl concentration was measured as described, with some modifications [31]. Briefly, a mixture of plasma sample, internal standard (IS, norfentanyl-D5), and acetonitrile was centrifuged, then the supernatant was evaporated to dryness under a nitrogen stream and reconstituted with the mobile phase, 0.1% (v/v) formic acid in water (solvent A)/0.1% (v/v) formic acid in acetonitrile (solvent B) (90:10). A Shimadzu HPLC system and LCMS-8045 (Shimadzu, Tokyo, Japan) were used and the analytical column (TSKgel ODS-100 V, 3 µm, 75 × 2.0 mm I.D., Tosoh, Tokyo, Japan) was maintained at 40ºC. The gradient was 0 min (10% solvent B)-2 (10%)-8 (70%)-8.01 (100%)-11 (100%)-11.01 (10%)-14 (10%), for 14 min per sample. Fentanyl and IS were monitored at 337.49/188.20 and 238.20/84.15, respectively.

Fentanyl in the formulation was measured by HPLC–UV. The formulation was soaked in methanol/acetonitrile (1:3) containing IS (papaverine) and sonicated at 36ºC, 38 kHz for 70 min. A Shimadzu HPLC–UV system was used with the analytical column (µBondasphere, PH 5 µm, 300 A, 150 × 3.9 mm, Waters Corporation, Milford, MA, USA) maintained at 40ºC, and isocratic mode was used in 0.1% (v/v) formic acid water/acetonitrile (70:30). UV absorption was monitored at 195 nm.

### Estimation of pharmacokinetic parameters

Pharmacokinetic parameters of fentanyl were estimated by model-independent moment analysis using Napp version 2.01 software [32]. Transdermal bioavailability (*F*) was calculated by dividing the area under the curve after transdermal administration (*AUC*_td,0-∞_) obtained from each animal by the mean *AUC* after intravenous administration (*AUC*_iv,0-∞_) corrected for the dose of fentanyl. The *k*_a_ of fentanyl administered transdermally was optimized by fitting data obtained after intravenous administration (elimination rate constant, *k*_e_; and volume of distribution at steady state, *Vd*_ss_) and transdermal administration (dose; concentration–time profile; and *F*) to a one-compartment model provided in the software. Fentanyl release ratio from the formulation (*F*_a_) and skin availability (*F*_skin_) were calculated as follows:

$${F}_{\text{a}}=\frac{{X}_{\text{unused}}-{X}_{\text{used}}}{{X}_{\text{unused}}}$$

$${F}_{\text{skin}}=\frac{F}{{F}_{\text{a}}}$$

where *X*_used_ and *X*_unused_ represent the residual amount of fentanyl in the formulation removed from the rats and the amount of fentanyl in the unused formulation, respectively.

### Statistical analysis

Patients were classified into two groups: those with a titration period within 5 days and those with a titration period of 6 days or more. Univariate logistic regression analysis was performed for gender, age, BMI, albumin concentration, initial dose, and comorbidities. The adjusted odds ratio (AOR) was calculated by multivariate logistic regression analysis for factors showing *p* < 0.1 in the univariate analysis. In the diabetic animal model study, the two groups were compared with an unpaired t-test. The analysis software was IBM SPSS ver. 27 (IBM, Armonk, NY, USA) and the significance level was set at *p* < 0.05.

## Results

### Relation between the titration period of transdermal fentanyl and DM as a comorbidity in the retrospective study

**Fig. 2:**
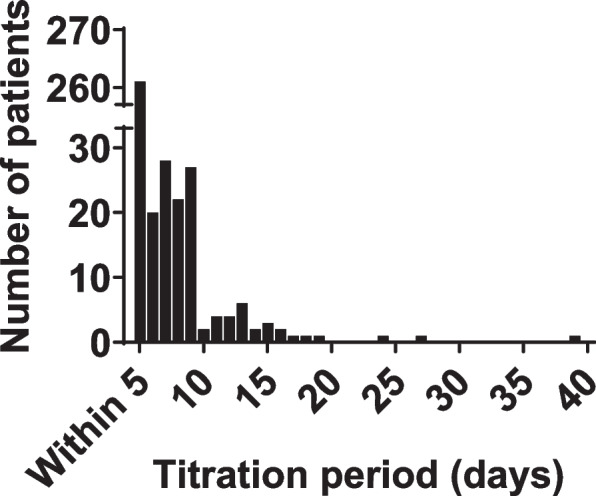
Titration period distribution of transdermal fentanyl in the retrospective study. The X-axis shows the titration period of transdermal fentanyl, and the Y-axis shows the number of patients by period (*n* = 387)

In the 387 patients analyzed (Fig. 1), the distribution of the titration period is shown in Fig. 2. Patients who showed the titration period of more than 5, 10, and 15 days amounted to 32.6%, 7.0%, and 2.1%, respectively, and the longest titration period was 39 days. Table 1 shows the patient backgrounds. The 150 comorbidities extracted are shown in Table S1. The most frequent cancer types involved the digestive organs (48.6%), lip, oral cavity and pharynx (18.1%), and respiratory and intrathoracic organs (12.7%), and there were only two non-cancer patients. The most frequent comorbidity groups were circulatory system diseases (41.1%), digestive system diseases (38.0%), and endocrine, nutritional and metabolic diseases (36.4%). DM was classified as type 2 (2.8%) or unspecified (17.1%).

**Table 1:** Patient backgrounds in the retrospective study

		Titration period	
	Total	Within 5 days	6 days or more
n	387	261	126
Male	239 (61.8)	158 (60.5)	81 (64.3)
Female	148 (38.2)	103 (39.5)	45 (35.7)
Age, years	66 [58–73]	66 [58–73]	66 [59–73]
Height, cm	162.3 [155.4–168.2]	162.0 [155.1–168.0]	162.4 [156.4–169.0]
Weight, kg	52.9 [45.7–60.7]	53.1 [45.7–61.6]	52.0 [45.7–59.0]
BMI, kg/m^2^	20.3 [18.3–22.5]	20.4 [18.4–22.9]	19.9 [18.2–21.8]
Serum albumin^a^, g/dL	2.9 [2.4–3.4]	2.9 [2.3–3.5]	2.9 [2.5–3.4]
Initial dose^b^, mg	1 [1–1]	1 [1–1]	1 [1–2]

Data are expressed as n (%) or median [25–75 percentile]

^a^ Data for 353 (within 5 days: 237 and 6 days or more: 116) patients

^b^ The 1 mg formulation contains 0.64 mg as fentanyl, and the estimated average absorption is 0.3 mg/day as fentanyl

In univariate analysis, no relation was observed between the titration period of 6 days or more and gender, age, BMI, serum albumin, or initial dose (Table 2). Twelve comorbidities with *p* < 0.1 in univariate analysis were included in the multivariate analysis, which confirmed that AOR for a titration period of 6 days or more was decreased in patients with unspecified DM (E14), AOR (95% confidence interval), 0.438 (0.217–0.884), while it was increased in patients with aortic aneurysm and dissection (I71), 4.590 (1.008–20.905).

**Table 2:** Logistic regression analysis related to the titration period of 6 days or more

	Univariate				Multivariate			
Factors	OR	95% CI		*p* value	AOR	95% CI		*p* value
Female	0.852	0.548	1.324	0.477	N/A			N/A
Age	1.007	0.989	1.025	0.460	N/A			N/A
BMI	0.955	0.897	1.016	0.146	N/A			N/A
Serum albumin^a^	0.977	0.723	1.320	0.879	N/A			N/A
Initial dose	1.029	0.771	1.375	0.845	N/A			N/A
Comorbidities^b^ (ICD-10 code)								
Malignant neoplasm of bladder (C67)	5.059	1.286	19.905	0.020	3.831	0.854	17.176	0.079
Secondary malignant neoplasm of other and unspecified digestive organs (C78.8)	3.554	0.836	15.113	0.086	2.009	0.401	10.056	0.396
Secondary malignant neoplasm of bone and bone marrow (C79.5)	1.584	0.938	2.677	0.085	1.563	0.890	2.745	0.120
Secondary malignant neoplasm of adrenal gland (C79.7)	3.779	1.085	13.159	0.037	2.475	0.610	10.047	0.205
Myeloid leukaemia (C92)	8.525	0.943	77.076	0.056	6.771	0.595	77.025	0.123
Other hypothyroidism (E03)	3.012	0.937	9.685	0.064	2.635	0.766	9.061	0.124
Unspecified diabetes mellitus (E14)	0.451	0.236	0.863	0.016	0.438	0.217	0.884	0.021
Unspecified dementia (F03)	8.525	0.943	77.076	0.056	8.415	0.882	80.257	0.064
Aortic aneurysm and dissection (I71)	4.300	1.057	17.485	0.042	4.590	1.008	20.905	0.049
Other diseases of digestive system (K92)	4.246	0.767	23.498	0.098	4.040	0.649	25.147	0.134
Other spondylopathies (M48)	4.300	1.057	17.485	0.042	3.055	0.652	14.322	0.157
Hyperplasia of prostate (N40)	4.246	0.767	23.498	0.098	4.241	0.672	26.765	0.124

*OR* Odds ratio, *AOR* Adjusted odds ratio, *CI* Confidence interval, *N/A* Not applicable

^a^ Data for 353 patients

^b^ Those showing *p* < 0.1 in univariate analysis

### Biochemical parameters, skin parameters, and intercellular lipids of SC in GK and Wistar rats

Non-fasting blood glucose level and body weight in GK rats (mean ± S.D., 437 ± 89 mg/dL and 339 ± 17 g) were significantly higher than those in Wistar rats (165 ± 29 mg/dL and 305 ± 10 g), respectively (n = 10, *p* < 0.05).

**Fig. 3:**
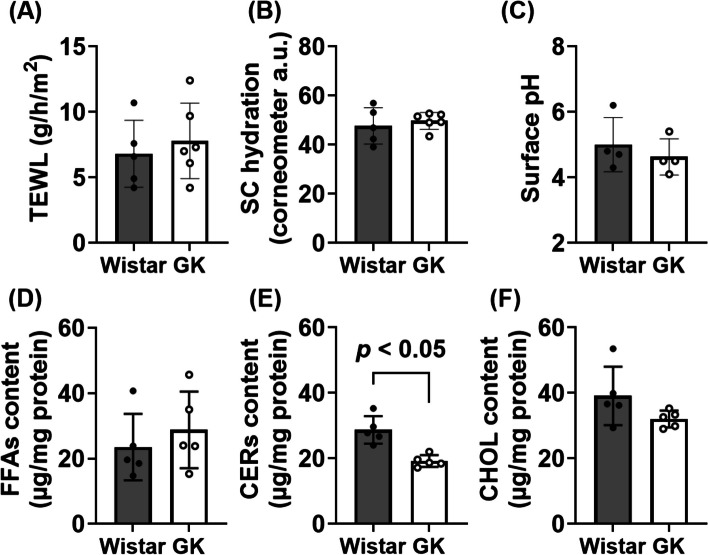
Skin parameters and intercellular lipids of SC in GK and Wistar rats. Transepidermal water loss (TEWL) (**A**), stratum corneum (SC) hydration (**B**), and surface pH (**C**) in the lateral abdominal skin were analyzed as skin parameters. As intercellular lipids of SC, the contents of free fatty acids (FFAs) (**D**), ceramides (CERs) (**E**), and cholesterol (CHOL) (**F**) were analyzed. Data are expressed as dots for individual values and bars for mean ± S.D. (*n* = 4–6)

No significant difference was observed in non-invasive skin parameters such as TEWL, SC hydration and surface pH between GK and Wistar rats (Figs. 3A-C). However, the composition of intercellular lipids of SC was significantly different, with the CERs content being approximately 30% lower in GK rats than in Wistar rats (Figs. 3D-F).

### Pharmacokinetic parameters of fentanyl after intravenous and transdermal administration in GK and Wistar rats

**Fig. 4:**
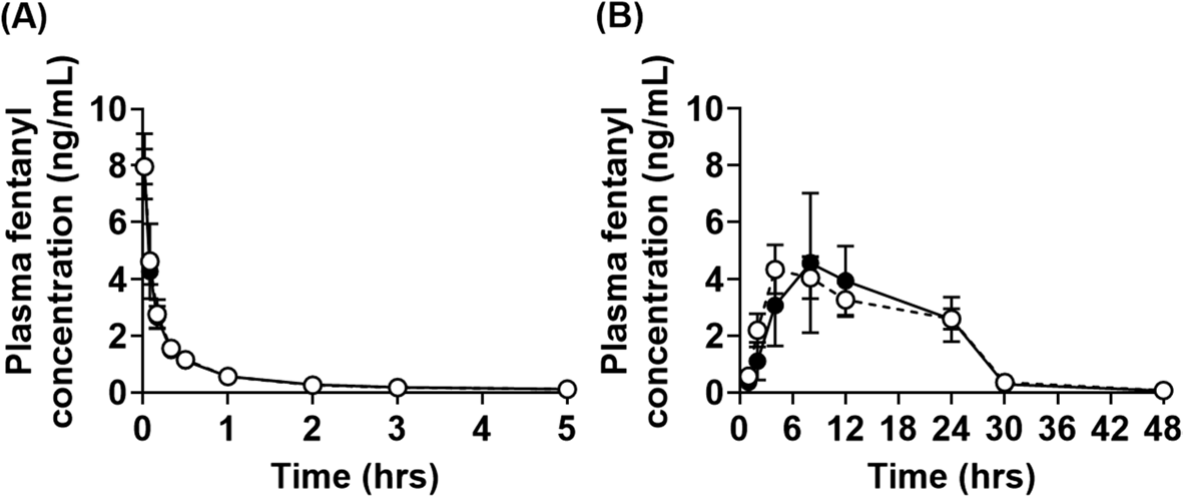
Plasma fentanyl concentration after intravenous and transdermal administration in GK and Wistar rats. Fentanyl citrate injection (10 µg/kg body weight as fentanyl) was administered intravenously to GK (open circle) and Wistar (closed circle) rats (**A**). Fentanyl citrate formulation (160 µg as fentanyl) was applied to the lateral abdominal skin and the formulation was removed at 24 h (**B**). Data are expressed as mean ± S.D. (*n* = 5)

Plasma concentration–time profile and pharmacokinetic parameters after intravenous administration of fentanyl (10 µg/kg) in GK and Wistar rats are shown in Fig. 4A and Table 3. After intravenous administration, no significant difference was observed in *AUC*_0-∞_, systemic clearance (*CL*_tot_), *k*_e_, or *Vd*_ss_ between GK and Wistar rats.

**Table 3:** Pharmacokinetic parameters of fentanyl after intravenous and transdermal administration in GK and Wistar rats

	Wistar rats	GK rats
Intravenous administration		
${AUC}_{0-\infty }$ (ng・hr/mL)	3.12 ± 0.16	3.22 ± 0.44
${CL}_{\text{tot}}$ (L/hr/kg)	3.21 ± 0.17	3.15 ± 0.42
${k}_{\text{e}}\times {10}^{-1}$ (/hr)	2.79 ± 0.26	2.55 ± 0.34
${Vd}_{\text{ss}}$ (L/kg)	6.49 ± 0.32	6.94 ± 1.77
Transdermal administration		
${C}_{\text{max}}$ (ng/mL)	4.74 ± 2.32	4.40 ± 0.86
${T}_{\text{max}}$ (hr)	9.60 ± 2.19	4.80 ± 1.79*
${AUC}_{0-\infty }$ (ng・hr/mL)	88.6 ± 27.5	87.9 ± 12.5
${CL}_{\text{tot}}/F$ (L/hr/kg)	6.51 ± 1.90	5.68 ± 0.81
${k}_{\text{a}}\times {10}^{-2}$ (/hr)	2.28 ± 0.67	3.17 ± 0.30*
$F$ (%)	53.0 ± 16.3	55.5 ± 7.1
${F}_{a}$ (%)	69.0 ± 6.3	78.0 ± 5.8*
${F}_{skin}$ (%)	76.3 ± 20.1	71.4 ± 10.5

Fentanyl citrate injection (10 µg/kg body weight as fentanyl) was administered intravenously to GK and Wistar rats. Fentanyl citrate formulation (160 µg as fentanyl) was applied to the lateral abdominal skin and the formulation was removed at 24 h. Data are expressed as mean ± S.D. (*n* = 5). * Significantly different from Wistar rats at *p* < 0.05

Plasma concentration–time profiles and pharmacokinetic parameters after transdermal administration of fentanyl (160 µg) in GK and Wistar rats are shown in Fig. 4B and Table 3. After transdermal administration, no significant difference was observed in peak concentration (*C*_max_), *AUC*_0-∞_, transdermal clearance (*CL*_tot_/*F*), *F*, or *F*_skin_ between GK and Wistar rats. However, in GK rats, the peak time (*T*_max_) was significantly shortened by approximately 50%, and *F*_a_ was significantly increased by approximately 10% compared to Wistar rats. The *k*_a_ in GK rats was significantly higher by approximately 1.4-fold than that in Wistar rats.

## Discussion

To prevent unrelieved pain and improve quality of life, it is important to consider not only the dose, but also the titration period in the dose titration of transdermal fentanyl. We hypothesized that skin changes due to comorbidities would affect the titration period of transdermal fentanyl. Many patients diagnosed with cancer also have DM [22, 23], and DM can alter skin properties [24, 26, 27, 33]. In this study, therefore, we especially focused on DM as a comorbidity and conducted a retrospective study and a diabetic animal model study.

In the retrospective study, the titration period of transdermal fentanyl was defined in terms of “dose changes” and “number of rescue opioids”, not pain intensity, and showed a large inter-individual variability (range: 5–39 days). This is consistent with a report that defined the time to achieve stable pain control with various opioids in terms of “pain intensity” and “number of rescue opioids”, obtaining a median time ranging from 4–22 days for mild to severe pain [34]. On the other hand, logistic regression analysis in the retrospective study indicated that the titration period was shorter in patients with unspecified DM. Possible reasons include effects on transdermal absorption due to changes of skin properties in DM [24] and on pain perception due to diabetic neuropathy [35], but it has not been established whether there is a causal relationship.

The retrospective study has a number of limitations, including lack of validation of the titration period evaluation criteria, the absence of plasma fentanyl concentration–time data, the entry of patients from a single center, the lack of adjustment for patient background factors, unspecified severity of comorbidities, and unknown effects of cancer chemotherapy on the skin. Additionally, since the time to reach steady state was estimated to be 5 days, the minimum period for follow-up, the effects on a shorter time scale could not be evaluated.

To test the hypothesis that skin changes due to DM would affect the titration period of transdermal fentanyl, GK rats were used as a diabetic animal model. The skin barrier function, measured in terms of TEWL, SC hydration and surface pH, differs in diabetic patients and diabetic models depending on the duration of DM onset and the severity of DM [24, 26, 27, 33]. In the GK rats used in this study, no changes in these parameters in the lateral abdominal skin were observed compared to Wistar rats, suggesting that the model in this study was an early-stage diabetic model.

The intercellular lipids of SC have a role as a skin barrier, and FFAs, CERs, and CHOL are present in approximately equimolar ratio [19]. We found that the CERs content was decreased in the lateral abdominal skin of GK rats compared to Wistar rats, whereas the levels of FFAs and CHOL were unchanged. In other diabetic animal models, OLETF rats and db/db mice, a decrease in relative mRNA expression of serine palmitoyl transferase, an enzyme related to CERs biosynthesis, was reported [26, 27]. On the other hand, a decrease of approximately 30% in CERs content has also been observed in atopic dermatitis (AD) [36]. Studies in model membranes that mimic AD indicate that the alteration in composition affects the lateral packing of lipids, the structure of drug permeation pathways and the permeability of ethyl *p*-aminobenzoate [37, 38]. Therefore, alterations in intercellular lipids of SC could affect the transdermal absorption of fentanyl.

In the pharmacokinetic experiments in rats, optimized *k*_a_ after transdermal administration was lower than *k*_e_ after intravenous administration, which is consistent with the observation of flip-flop kinetics in humans [13–16]. Generally, the steady state is reached in about five times the half-life, which is dependent on the elimination half-life of the drug and is inversely proportional to *k*_e_. However, in the case of very slow absorption from the administration site, so-called flip-flop kinetics may be observed in which the apparent terminal half-life is dependent on *k*_a_. Under this condition (*k*_a_ < *k*_e_), the half-life is inversely proportional to *k*_a_. In GK rats, *k*_a_ was significantly higher and *T*_max_ was significantly shorter after transdermal administration compared to Wistar rats, although no difference was observed in *CL*_tot_ after intravenous administration. These results suggest that fentanyl rapidly reaches the steady state during repeated transdermal administration in DM and support the findings in the retrospective study in humans.

To investigate the steps that affect the transdermal absorption of fentanyl in GK rats, *F*_a_ and *F*_skin_ were calculated. *F*_a_ is commonly used in the assessment of transdermal fentanyl, reflecting distribution from the formulation to the SC [4, 5, 39–41]. On the other hand, *F*_skin_, the fraction absorbed through the skin, could be affected by reservoir effects, binding, and metabolism in the skin [42–45]. The higher value of *F*_a_ in GK rats compared to Wistar rats, despite the lack of difference in *F*_skin_, suggests that the decrease of CERs content in GK rats was one of factors contributing to an increase in the distribution of fentanyl from the formulation to the SC.

A further possible issue is that DM patients may be using skincare products containing CERs to improve their skin condition. This might increase the content of CERs in the SC, and at least partially reverse the increase of *k*_a_ and the shortened titration period of fentanyl after transdermal administration. However, the effects of supplementing CERs on transdermal absorption of fentanyl are currently unknown, and further investigation is needed.

This study focused on type 2 DM, because no patient with type 1 DM was identified in this retrospective study and because type 2 DM accounts for approximately 90% of all DM [24, 46]. However, the prevalence of type 1 DM is increasing [46], and skin disorders are observed in type 1 DM as well as type 2 DM [47]. Thus, type 1 DM may also affect the transdermal absorption of fentanyl, though further investigation will be needed to confirm this.

A shorter titration period of transdermal fentanyl would contribute to improving patients’ quality of life. However, in clinical practice the dose is titrated after an interval of several days, because a long time is required to reach the steady state. Our findings indicate that the steady state is reached earlier in DM, so that it may be feasible to carry out pain assessment and dose adjustment of fentanyl at an earlier time point in cancer patients with DM as a comorbidity.

## Conclusion

Our results indicate that transdermally administered fentanyl reaches the steady state earlier in DM. For rapid pain relief in cancer patients with DM, transdermal fentanyl dose adjustment of fentanyl may be feasible at an earlier time point than in cancer patients without DM.

## Supplementary Information

Supplementary Material 1.

## Data Availability

The data analyzed in this study are available from the corresponding author on reasonable request.

## Abbreviations

AD: Atopic dermatitis
AUC: Area under the curve
AOR: Adjusted odds ratio
BMI: Body mass index
CERs: Ceramides
CHOL: Cholesterol
CI: Confidence interval
CYP: Cytochrome P450
*C*_max_: Peak concentration
*CL*_tot_: Systemic clearance
*CL*_tot_/*F*: Transdermal clearance
DM: Diabetes mellitus
ESMO: European Society for Medical Oncology
*F*: Transdermal bioavailability
*F*_a_: Fentanyl release ratio from the formulation
FFAs: Free fatty acids
*F*_skin_: Skin availability
GK rats: Goto-Kakizaki rats
HPLC: High-performance liquid chromatography
ICD-10: International Classification of Diseases 10th Revision
IS: Internal standard
*k*_a_: Absorption rate constant
*k*_e_: Elimination rate constant
OLETF rats: Otsuka Long-Evans Tokushima Fatty rats
OR: Odds ratio
SC: Stratum corneum
TEWL: Transepidermal water loss
*T*_max_: Peak time
*Vd*_ss_: Volume of distribution at the steady state
